# Pamphlets Received

**Published:** 1881-06-20

**Authors:** 


### *PAMPHLETS RECEIVED.*

HOW WE FED THE BABY to Make it Healthy and Happy, with Health Hints; by C. E. Page, M. D. New York, Fowler & Wells, publishers, 753 Broadway.

CLINICAL ILLUSTRATIONS OF FAVUS, and its treatment by a New Method of Depilation; by L. Duncan Bulkley, A. M., M. D., attending physician for Skin and Venerial diseases at the New York Hospital, out-patient department; late physician to the Skin Department, Demilt Dispensary, New York, etc. [Reprinted from the Archives of Dermatology, Vol. vii, No. 2, April, 1881.] New York, G. P. Putnam's Sons, 27 and 29 West 23d street, 1881.

EIGHTH BIENNIAL REPORT of the Trustees, Superintendent and Treasurer of the Illinois Asylum for Feeble-Minded Children at Lincoln. October 1, 1880. Springfield, H. W. Rokker, State Printer and Binder, 1881.

REPORT OF THE PENNSYLVANIA HOSPITAL FOR THE INSANE, for the year 1880; by Thomas S. Kirkbride, M. D., Physician-in-Chief and Superintendent. Published by order of the Board of Managers. Philadelphia, 1881.

